# Evaluation of Morphine with Imiquimod as Opioid Growth Factor Receptor or Nalmefene as Opioid Blocking Drug on Leishmaniasis Caused by *Leishmania major* in Vitro

**Published:** 2019

**Authors:** Javad JABARI, Fatemeh GHAFFARIFAR, John HORTON, Abdolhosein DALIMI, Zohreh SHARIFI

**Affiliations:** 1. Department of Parasitology, Faculty of Medical Sciences, Tarbiat Modares University, Tehran, Iran; 2. Tropical Projects, Hitchin, UK; 3. Blood Transfusion Research Center, High Institute for Research and Education in Transfusion Medicine, Tehran, Iran

**Keywords:** *Leishmania major*, Morphine, Imiquimod, Nalmefene, Apoptosis

## Abstract

**Background:**

In this research, the effect of morphine on promastigotes and amastigotes of *Leishmania major* has been investigated in the presence of nalmefene as a blocking opioid drug and imiquimod as an opioid growth factor receptor.

**Methods:**

This study was conducted at Tarbiat Modares University, Tehran, Iran in 2015–2018. Morphine with different concentration (0.1, 1, 10 and 100 1μg/ml) alone and with imiquimod (0.01, 0.1 and 1μg/ml) and nalmefene (0.1, 1 and 10 μg/ml) on promastigotes and amastigotes in macrophages and also the percentage of infected macrophages was investigated. For evaluation of the apoptosis, we used flow cytometry method. The effect of imiquimod and nalmefene on glucantime and amphotericin B as current drugs for treatment of leishmaniasis was evaluated too.

**Results:**

The effect of morphine on promastigotes and amastigotes has a reverse relationship with its concentration. The results of flow cytometry for drug-treated promastigotes revealed that apoptosis and necrosis did not increase markedly relative to the control group. A combination of morphine and imiquimod in concentrations of 0.05, 5 and 5 μg/ml had a pronounced effect on reduction and prevention of macrophage infection with amastigotes. Morphine at a concentration of 0.1 μg/ml plays the role of adjunctive treatment. In amastigote assay we found the better results in group that get glucantime 25 μg/ml+ imiquimod 0.5 μg/ml.

**Conclusion:**

This effect is strengthened with imiquimod and weakened with nalmefene. Using high dose morphine and nalmefene had reverse effects. They suppress immune system and had no controlling effect in macrophages amastigote infection and reduction of promastigotes.

## Introduction

*Leishmaniasis* is caused by various species of *Leishmania*. It is a serious public problem, specifically in developing countries. At least 350 million people in 98 countries are at risk of self-limiting skin lesions with possibility of involvement of internal organs. Unfortunately, no effective vaccine or drug is available to control the disease. Moreover, existing drugs such as pentavalent antimony are toxic and their use may also lead to relapse and drug resistance. Therefore, we are in urgent need for new and effective treatment methods. One approach may be to apply the concurrent effects of two or more drugs (1–3). In addition to new drugs as specific treatment, the identification of drugs that can act as adjunctive therapy, as with treatment for malignancies, is a potential approach that could improve efficacy and reduce toxicity.

Opioid immune modulator effects on different infections have been demonstrated. Morphine might affect human lymphocyte responses while its effect on neuronal function was earlier known. This finding paved the way for more studies on neuropeptides and led to discovery of some neuropeptide receptor effects on the immune cells. Neuropeptides affect immune system reactions. Such effects are transferred by the opioid receptors as intra-membrane paired receptors of G protein. Morphine and other opioids begin a cascade of effects from the cell itself. They cause changes in the normal cell functions of the immune elements. The effects are most obvious in macrophages and lymphocytes (4, 5).

Morphine may increase immune defensive responses in the host, which in turn controls the infection (6, 7). These effects are dose-dependent. Treatment with low dose morphine causes protection and defense against infections (8–10), while high dose or long-term treatment suppresses immune responses (11). Reactivated nitrogen intermediates are defensive mechanisms against *Leishmania* and morphine adjusts iNOS, as a result of NO secretion (12, 13).

Opioids provide immune effects by direct and indirect mechanisms. Direct effects are rooted inactivation of opioid receptors by immune cells and lead to a variety of changes in their physiologic functions. On the other hand, the central nervous system mediates indirect effects of opioids.

Experiments reveal that chronic consumption of morphine weakens normal function of immune cells, specifically of macrophages and lymphocytes (5, 13, 14). Patients who receive chronic opioid treatment and those addicted to opioids experience clinical changes in their immune functions, which make them more vulnerable to infection (14, 15). Despite many reports of negative effects of morphine on the immune system, stimulator effects of the morphine on the system have also been reported (4).

Morphine may increase defensive immune responses in the endogenous and exogenous forms, and weaken the infection (6–10, 16). Immune system does not operate alone and is also affected by other organs, specifically by the central nervous and neuroendocrine systems. In addition, immune system may mutually affect the function of these systems (17).

The morphine effects on imiquimod as an opioid growth factor receptor (OGFr) stimulant or on nalmefene as a blocker of opioid receptor have not previously been studied in *leishmaniasis*. In addition, the assessment of their receptor and blocker effects on the efficacy of glucantime, a pentavalent antimony compound and amphotericin-B used for treatment and have not been investigated.

## Materials and Methods

This study was conducted at Tarbiat Modares University, Tehran, Iran in 2015–2018.

It was approved by Ethical Committee of Faculty of Medical Sciences, Tarbiat Modares University, NO 52D/ 8181 date 22 Feb 2015.

### Preparation and collection of parasites

The study used *L. major* (MRHO/IR/75/ER), a strain derived from *L. major* maintained by the Parasitology Department at Tarbiat Modares University. The nutrient culture media RPMI1640 was purchased as a prepared solution from Gibco, France. In order to prevent bacterial infection, each ml of culture media received 100 units/ml penicillin and 100 mg/ml streptomycin.

### Drugs preparation

We used sulfated morphine (Temad Co, Teheran, Iran) which was then diluted for use at concentrations of 0.1, 1, 10 and 100 1μg/ml. Imiquimod (Invivogen, Toulouse, France) produced in a special solvent by the manufacturing company was used at concentrations of 0.01, 0.1 and 1μg/ml. Nalmefene hydrochloride dehydrate (Selincro, France) was diluted in DMSO to give concentrations of 0.1, 1 and 10 μg/ml; amphotericin B (GILEAD UK) was used at concentrations of 0.5 and 1μg/ml; and glucantime (Sanofi-Aventis France) was used at concentrations of 12.5, 25 and 50 μg/ml.

### Promastigote assay

Promastigote numbers were measured using a hemocytometer (Neubauer Chamber). The number of parasites was counted before treatment and after treatment with various drug doses with non-treated promastigotes as a control group. Promastigote counting was done three times at 24, 48 and 72 h after incubation with drugs. In the negative control group, promastigotes were cultured without adding the appropriate drug solvent without drug. All experiments were conducted in triplicate.

### Evaluation of promastigote and macrophage viability by MTT

The MTT method was used to evaluate the cytotoxic effects of morphine, imiquimod and nalmefene on promastigote viability. In this test, 2×16^6^ promastigotes exposed to the drugs in various concentrations are first counted after 24, 48 and 72 h. Then, 100 μl of the sample is put in triplicate, on the plate and 20 μl MTT reagent (with the ultimate concentration of 0.5 μg/ml) is added. The plates are incubated for 3–5 h at 21 °C. The same is repeated for macrophage viability, but the plates are incubated at 37 °C. Plates are then centrifuged for 10 min at 2000 rpm. The supernatant is discarded and 100 μl DMSO is added into each well.

After 15 min, light absorption at 570 nm was measured by ELISA reader. Viability of promastigotes was calculated using the following formula:

$$\text{Cell}\;\text{Viability}\;(\%)=\frac{\text{Drug}\;\text{well}\;\text{absorption}-\text{Blank}\;\text{well}\;\text{absorption}\times 100}{\text{Control}\;\text{well}\;\text{absorption}-\text{Blank}\;\text{well}\;\text{absorption}}$$

### Amastigote assay

To evaluate effects on amastigotes, we firstly cultured J774 macrophages in a RPMI-1640 media containing 100 IU/ml penicillin, 100 μg/ml streptomycin and 10% FCS. Each well of the 12-cell culture plate had a sterile coverslip in the bottom and 1ml culture media containing 10^5^ macrophages was added. The plate was then incubated in 37 °C with 5% CO_2_. To infect macrophages, 1 ml culture media containing 10^6^ promastigotes at their static stage were added to the wells and the plate incubated at 37 °C with 5% CO_2_. Six hours later, the supernatant is discarded and new culture media is added, to remove non-adhesive macrophages to the well bottom and non-entered promastigotes into the cell.

An inverted microscope was used to confirm infection of macrophages with parasite isolates. Twenty four hours after the primary culture; drug compounds were added. Control well contains parasite-infected macrophage without drug. Each sample was treated triplicate. The infected macrophages with and without (control group) various drug concentrations were incubated for 24, 48 and 72 h. Then the coverslips were removed, stained with Giemsa, and amastigotes within the macrophages counted by optical microscope.

### Evaluation of Apoptosis with Flow cytometry

Flow cytometry is a method for evaluating the phenotype and cell characteristics. Annexin V-FITC Apoptosis detection kit- (Bio vision USA) was used for flow cytometry. Promastigotes exposed to different concentrations and various drug compounds with appropriate controls were collected after 24, 48 and 72 h incubation into 1.5 ml microtubes and centrifuged for 5 min at 3000 rpm. The supernatant was discarded and 500 μl binding buffer was added to the sediment, followed by 5 μl oncesin and 5 μl propidium iodide. Samples were incubated for 5 min in darkness at room temperature. Absorption of annexin-v by cells was assessed by FACSCaliber (BD Biosciences) and the results were analyzed using Cellquest Software.

## Results

### Promastigote Assay

**Fig. 1:**
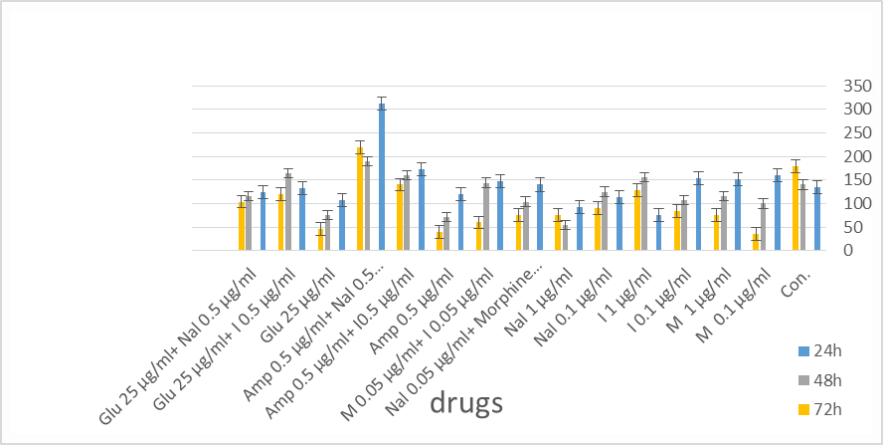
Mean and SD of the number (×10^4^) of promastigotes treated by various concentration of drugs and control group after 24, 48 and 72 h. CON: Control; M: Morphine; I: Imiquimod; Nal: Nalmefene; Amp: Amphotericin B; Glu: Glucantime

The highest meaningful difference from the control group occurred after 72 h. The greatest differences were seen with amphotericin B at both concentrations used and with morphine 0.1 μg/ml. There was no significant difference from control when nalmefene 1μg/ml was added to amphotericin 0.5 μg/ml (Fig. 1).

### Promastigote and macrophage viability by MTT

Most drugs or drug combinations were significantly different from the control group. Amphotericin 0.5 μg/ml with either imiquimod 0.5 μg/ml, or nalmefene 0.5 μg/ml were not different from the control group, the number of live macrophages in the culture being close to that of control group without drug (Table 1).

**Table 1:** Viability of drug-treated promastigotes in comparison with the control group using MTT results

***Groups***		***Percentage of viability of promastigotes after different times***		
		24h	48h	72h
1	Con.	100	100	100
2	M 0.1 μg/ml	100	56	21
3	M 1 μg/ml	90	66	42
4	I 0.1 μg/ml	69	59	47
5	I 1 μg/ml	54	67	72
6	Nal 0.1 μg/ml	62	68	51
7	Nal 1 μg/ml	50	30	45
8	Nal 0.05 μg/ml+ M 0.05 μg/ml	64	55	45
9	M 0.05 μg/ml+ I 0.05 μg/ml	100	80	35
10	Amp 0.5 μg/ml	68	40	24
11	Amp 0.5 μg/ml+ I 0.5 μg/ml	100	88	77
12	Amp 0.5 μg/ml+ Nal 0.5 μg/ml	100	100	100
13	Glu 25 μg/ml	66	43	28
14	Glu 25 μg/ml+ I 0.5 μg/ml	75	92	68
15	Glu 25 μg/ml+ Nal 0.5 μg/ml	64	56	60

CON: Control; M: Morphine; I: Imiquimod; Nal: Nalmefene; Amp: Amphotericin B; Glu: Glucantime

### Amastigote Assay

The number of amastigotes was reduced inside the macrophages in all treatment groups. There appeared to be a significant difference between the treatment and control group. The difference shows the positive inhibitory effect of drugs on macrophages. In the group with glucantime (25 μg/ml) + imiquimod (0.5 μg/ml) the number of amastigotes was reduced to zero (Table 2).

**Table 2:** Mean and SD of amastigote numbers in each drug-treated macrophage and control group, and percent of infected macrophages after 72 hours (two separate experiments)

***Groups***	***amastigotes number per macrophages Mean ±SD***
Con.	4.29±0.72
M 0.1 μg/ml	0.5±0.14
M 1 μg/ml	1.36±0.33
I 0.1 μg/ml	0.07±0.042
I 1 μg/ml	0.115±0.04
Nal 0.1 μg/ml	2.32±0.45
Nal 1 μg/ml	1.24±0.056
Nal 0.05 μg/ml+ M 0.05 μg/ml	0.18±0.08
M 0.05 μg/ml+ I 0.05 μg/ml	0.025±0.02
Amp 0.5 μg/ml	0.07±0.04
Amph 0.5 μg/ml+ I 0.5 μg/ml	0.04±0.028
Amp 0.5 μg/ml+ Nal 0.5 μg/ml	0.25±0.09
Glu 25 μg/ml	0.065±0.03
Glu 25 μg/ml+ I 0.5 μg/ml	0
Glu 25 μg/ml+ Nal 0.5 μg/ml	0.04±0.028

CON: Control; M: Morphine; I: Imiquimod; Nal: Nalmefene; Amp:Amphotericin B; Glu: Glucantime

**Fig. 2:**
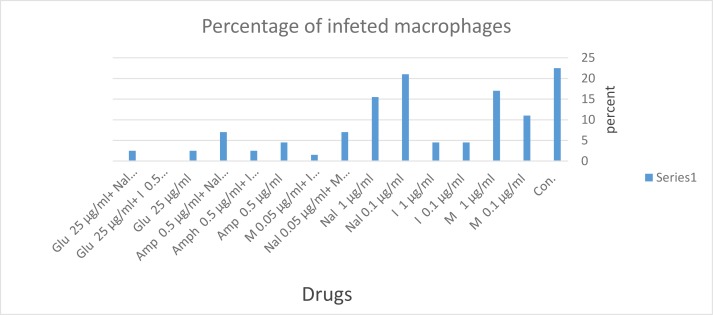
The percentage of infected macrophages treated with different drugs and on control group. CON: Control; M: Morphine; I: Imiquimod; Nal: Nalmefene; Amp: Amphotericin B; Glu : Glucantime

The percentage of infected macrophages show in Fig. 2.

### Flow Cytometry Results

**Fig. 3:**
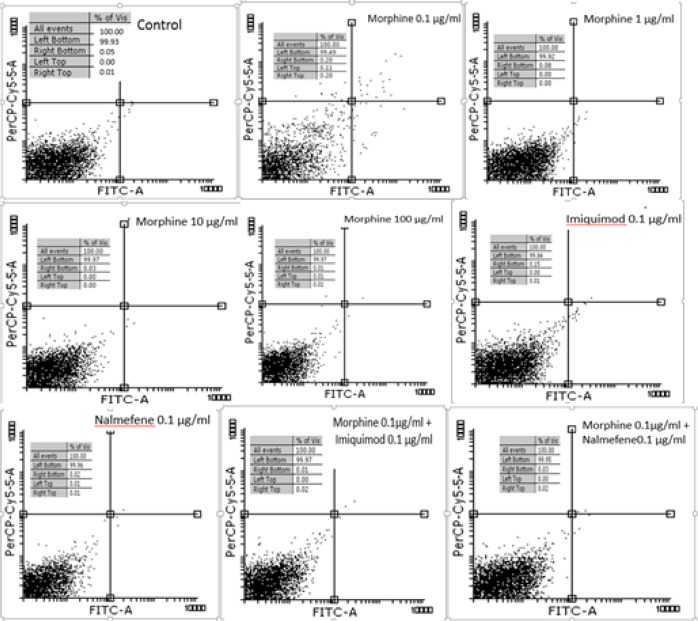
Results of flow cytometry for drug-treated promastigotes and control group after 24 hours. Left bottom alive promastigotes; right bottom apoptosis promastigotes; right top: delayed apoptosis promastigotes; and left top: necrosis promastigotes

Based on flow cytometer results, the proportion of apoptosis delayed apoptosis and necrosis was very low in all treated groups after 24 h. The drugs have no toxic effect on promastigotes after 24 h and were not significantly different from control (Fig. 3).

## Discussion

The promastigote assay of morphine provides the best result after 72 h for all concentrations and specifically for the lowest concentration of 0.1 μg/ml of morphine. Amphotericin B revealed good results by itself, however, combination of amphotericin B and imiquimod and especially nalmefene increases promastigote numbers. With nalmefene 0.05 μg/ml and amphotericin 0.5 μg/ml, the number of promastigotes increased 24, 48 and 72 h after drug treatment. Glucantime was also more effective at reducing promastigote numbers by itself compared to its combination with imiquimod or nalmefene. With other treatments, we observed a reduction of promastigote numbers.

In the amastigote assay, the best outcome occurred with a morphine concentration of 0.1 μg/ml, while an increase in concentration reduces the effect and the highest concentration is similar to the control group. The best result for imiquimod also relates to the lowest concentration, while with nalmefene at a low concentration showed no effect and higher concentrations had a limited effect.

Morphine and nalmefene at lower concentrations were more effective and increase of concentration reduced the effectiveness since nalmefene at lower concentrations is not able to block all receptors. Combination of morphine 0.5 and 5.0 μg/ml; with nalmefene 0.5 and 5 μg/ml has similar effect to that of control, in relation to macrophage infection inhibition. This demonstrates the inhibition effect of nalmefene on opioid receptors. Morphine 0.05 μg/ml and nalmefene 0.05 μg/ml had little effect on prevention of macrophage infection.

Imiquimod and morphine in concentrations of 0.05 μg/ml and 0.5 and 5 μg/ml had a considerable effect on reduction and prevention of macrophage infection with amastigotes. This shows the effect of imiquimod as a stimulant and augmenter of opioid receptors.

Amphotericin B results are good when it is used alone and slightly better when it is used with imiquimod, but became less effective when it was combined with nalmefene. Amphotericin in concentration 0.5 μg/ml has positive effect on preventing macrophage infection. Amphotericin 0.5 μg/ml combined with imiquimod 0.5 μg/ml improved the results slightly while with nalmefene, an increase of macrophage infection may relate to blocking of opioid receptors and limiting the penetration of amphotericin.

Similarly, glucantime by itself and when used with imiquimod destroyed amastigotes, but the effects were reversed with nalmefene. Again, one may suggest that glucantime uses opioid receptors to enter into macrophage.

Glucantime 25 μg/ml had appropriate effect on reduction of macrophage infection. The greatest effect on amastigote infected macrophages was achieved with a combination of glucantime 25 μg/ml and imiquimod 0.5 μg/ml, with apparent elimination of macrophage infection. Imiquimod increases the effect of morphine and glucantime, while it has no effect on amphotericin B.

The number and proportion of amastigote-infected macrophages are high with all nalmefene concentrations, suggesting that the drug is not effective in infection control. Nalmefene in low concentration has little effect on morphine and this effect reduces as concentration increases. Nalmefene has a reverse effect on amphotericin B, while it had little or no effect on glucantime.

Imiquimod with concentration of 1 μg/ml had little effect on promastigotes, as promastigote numbers increased over three consecutive days. However, it may have positive effect on hosts, through immune system strengthening and increase of receptors.

Imiquimod in concentration of 0.01 μg/ml has better effects on macrophage infection control relative to concentrations of 0.1 and 1 μg/ml. Moreover, flow cytometry results reveal that apoptosis and necrosis production is low. Therefore the effects of this drug relate more to augmentation of the immune system and expression of cell level receptors, rather than producing apoptosis or necrosis.

Nalmefene controls opioid receptors. It binds strongly to three opioid receptors, as a result of which all opioid receptors are blocked. Nalmefene increases intracellular pathogenicity through inhibition of opioid receptors (18, 19). Opioid growth factor (OGF) does not destroy cancer cells and is not cytotoxic. However, it prevents cancer cell growth and provides immune mechanisms with the potential to destroy cancer cells. OGF also works in harmony with cancer chemotherapy, so that the drug effects are greater among those who receive a combination of OGF and chemotherapy drugs (20).

Imiquimod augments TLR7, which exists on macrophage and dendritic cells (20). TLR7 can direct type Th-1 immune reactions. Such responses are required for degradation and destruction of *Leishmania* parasites (21). Imidazkevinolin compounds such as imiquimod and resiquimod change the immune response through strengthening anti-virus and anti-tumor properties (22–24). Imiquimod (Aldara, R837, S-26308) is the most widely used and the most effective drug for treatment of genital and anal external warts, basal cell carcinoma, Kaposi cancer, chronic hepatitis type C and intraepithelial carcinoma. Although imiquimod has been used successfully in animals, including treatment of many tumors such as melanoma, lung sarcomas and breast cancer, imiquimod's functional mechanism remains unknown (25,26). It acts as an agonist of RLR-7 (27) inducing its anti-tumor effects through immune responses and stimulation of apoptosis (28). Imiquimod induces its effects through intermediates such as TNFa, IL-1a and IL-12 (21–24).

Imiquimod, is a synthetic imiquinolin connected to TLR7 and used to treat some cancers including surface basal cell carcinoma and its purity degree reported from 79% to 82%. Moreover, it has anti-cancer, immune stimulating and immune regulating effects. It is used to treat some autoimmune diseases in human such as multiple sclerosis, Behcet's syndrome and optic neuritis. Moreover, it has been used in some cases to treat AIDS successfully (26, 29, 30).

## Conclusion

Overall this work suggests that morphine at a concentration of 0.1 μg/ml plays the role of an adjunctive treatment, which enhances the immune system in infection control. This effect is strengthened with imiquimod and weakened with nalmefene. Using high dose morphine and nalmefene had reverse effects. They suppress immune system and had no controlling effect in macrophages amastigote infection and reduction of promastigotes.
